# Study of P21 Expression in Oral Lichen Planus and Oral Squamous Cell Carcinoma by Immunohistochemical Technique

**Published:** 2015-09

**Authors:** Fahimeh Baghaei, Setareh Shojaei, Noushin Afshar-Moghaddam, Massoumeh Zargaran, Verisheh Rastin, Mohsen Nasr, Abbas Moghimbeigi

**Affiliations:** 1 Dept. of Oral and Maxillofacial Pathology, School of Dentistry, Hamadan University of Medical Sciences, Hamadan, Iran.; 2 Dept. of Pathology, Schools of Medicine, Isfahan University of Medical Sciences, Isfahan, Iran.; 3 Dental Research Center, Dept. of Oral Maxillofacial Pathology, School of Dentistry, Hamadan University of Medical Sciences, Hamadan, Iran.; 4 Dept. of Oral and Maxillofacial Pathology, School of Dentistry, Kurdestan University of Medical Sciences, Sanandaj, Iran.; 5 Dept. of Pathology, Alzahra Hospital, Isfahan University of Medical Sciences, Isfahan, Iran.; 6 Dept. of Biostatistics and Epidemiology, School of Public Health, Hamadan University of Medical Sciences, Hamadan, Iran.

**Keywords:** Oral squamous cell carcinoma, Oral lichen planus, P21

## Abstract

**Statement of the Problem:**

Lichen planus is a mucocutaneous disease that is relatively common in middle aged individuals. Some studies have shown that oral lichen planus has a potential to progress to squamous cell carcinoma.p21 is a cyclin-dependent kinase inhibitor that regulates the cell cycle, thus it acts as an inhibitor in cell proliferation.

**Purpose:**

This study was aimed to evaluate and compare the immunostaining of p21 (as a proliferation inhibitory factor) in oral lichen planus (OLP) and oral squamous cell carcinoma (OSCC).

**Materials and Method:**

In this descriptive cross-sectional study, p21expression was investigated in 24 samples of oral lichen planus (OLP), 24 samples of oral squamous cell carcinoma (OSCC) and 24 samples of oral epithelial hyperplasia (OEH) by employing immunohistochemical staining.

**Results:**

The mean percentage of p21-positive cells in OSCC (54.5±6.6) was significantly higher than that in OLP (32.8±6.08) and OEH (9.4±3.8). Moreover, OLP samples expressed p21 significantly higher than the OEH. Kruskal Wallis test revealed a statistically significant difference between the groups regarding the intensity of staining (*p*< 0.001).

**Conclusion:**

According to the findings of this study, the expression of p21 might be related to the potential carcinogenic transformation of lichen planus to SCC. Therefore, continuous follow-up periods for OLP are recommended for diagnosis of the malignant transformations in early stages.

## Introduction

Initially described by Erasmus Wilson in 1896, lichen planus is a relatively common chronic skin disease and one of the most common oral mucosal diseases.[1] It is an autoimmune skin and mucosal disorder mediated by T-lymphocytes.[2] Some studies have shown that oral lichen planus (OLP) has the potential to develop to oral squamous cell carcinoma (OSCC).[3-5] However, the relation between OLP and OSCC is even now controversial since many researchers believe that still there is not enough data to prove the correlation between OLP and occurrence of cancer.[6] OSCC comprises approximately 90% of oral cancers and is the eleventh most common cancer of human, showing an increased incidence. Despite the current improvements in the treatment of this disease, OSCC still has a high mortality rate and its 5-year survival rate is 45-50%. OSCC has different etiological factors; nonetheless, no single causative factor is clearly defined or accepted.[1, 3-4,6]

A cancer occurs when mutation or amplification causes dysregulation of the genes that control the cell cycle.[7] The main targets of genetic damage that leads to cancer are four groups of the normal cell cycle regulatory genes; including 1-proto-oncogenes, which accelerate the growth, 2- tumor suppressor or growth inhibitor genes, 3-genes that regulate the apoptosis, and 4- genes that are involved in DNA repair.[7] Abnormalities in oncogenes and tumor suppressor genes have been detected in oral carcinoma.[1] Different stages of cell cycle are controlled and conducted by cyclins, cyclin-dependent kinases (CDK), and their inhibitors.[7-8] p21 protein is a potent CDK inhibitor belonging to the tumor suppressor genes. It binds to CDK and inhibits the RB (retinoblastoma) phosphorylation and the activity of the CDK2 and CDK4 complex which is essential for the cells to enter G1phase. Therefore, it is recognized as a regulator of cell-cycle progression in G1 phase.[7-8] The gene mutations that involve the CDK complex are identified in some malignant neoplasms and are likely to act in favor of cell proliferation process.[7]

Only few studies have evaluated and compared the immunostaining of proteins (such as p21) that control the cell cycle in OLP and OSCC. This study was aimed to evaluate and compare the incidence of p21 (as a proliferation inhibitory factor) in OLP and OSCC. 

## Materials and Method

The samples of this descriptive – analytic study were collected from the archives of the Department of Pathology, School of Dentistry Isfahan and Hamadan, and Isfahan Alzahra Hospital. A total of 72 samples consisting of 24 paraffin blocks from each of the three lesions; OLP, OSCC, and oral epithelial hyperplasia without dysplasia (OEH) as the control group were recruited. All the samples were reviewed by a pathologist concerning the criteria described in the related articles.[9-10] Histopathological diagnostic criteria described by Eisenberg, including basal cell hydropic degeneration, band-like lymphocytic infiltrate at epithelial-stromal junction, normal epithelial maturation pattern were considered for diagnosis of oral lichen planus.[10] Cases which were exposed to risk factors such as smoking/drinking behaviors or with dysplastic changes were excluded. Moreover, the patients' medical records should have documented no history of taking medications.

Any sample with improper fixation or with suspicious diagnosis or necrotic tissue was excluded. 15 SCC samples had a moderate to good differentiation and the rest were moderate to poorly differentiated.

The 4-μm paraffin sections were prepared and placed on poly-l-lysine coated slides. Having been cut, the slides were placed in an oven with a temperature of 58˚C for 24 hours. The slides were then dried and a 3% peroxidase solution was poured on each of them. The slides were stored in a dark and damp room for 10 minutes and then rinsed with PBS for 5 minutes. The primary monoclonal antibody (Monoclonal antibody; code PM 354 AA, Bio care, USA) was poured on slides and the staining process was performed based on the instruction provided by manual of BIO CARE company in 2013. After being stored in dark and damp room at room temperature, the slides were rinsed in separate containers of PBS for 5 minutes. The secondary antibody solution was poured onto slides and they were incubated with super enhancer at room temperature for 30 minutes. 

In the polymer phase, the slides were incubated with polymer-HRP (SS-Label) at room temperature. In chromogenic phase, one or two drops of DAB solution (3, 5 Diamino Banzidin 1:20) were added for 5 minutes. The slides were then rinsed with PBS and distilled water respectively for 2 minutes. Hematoxylin staining was performed within 5 minutes; the slides were then placed in lithium carbonate for 2 minutes and then rinsed with tap water for 5 minutes. The slides were soaked in 96% and 100% alcohol and xylene for 5 minutes; the samples were then mounted. The stained slides were studied under 100 x and 400 x light microscope (Olympus CX31; Tokyo, Japan). They were compared with the positive control sample (melanoma) to verify the accurate staining.

The cells with brown-stained nucleus (with any intensity) were considered as positively stained cells. In each slide, a total of 1000 cells were counted (under 400 x) in areas with higher p21 staining. The total number of stained cells per 1000 cells was calculated in percent. In the current study, the amount of stained cells in each lesion was scored based on the study published by Agarwal *et al*.[11] as (+3) 50 %<, (+2) 30-50%, (+1) 10-30%, and (negative) 10%>.

Cells staining intensity was determined using modified quick score method[12] as negative (no staining), weak, moderate, and strong. Data were analyzed by SPSS software, version 13. The ANOVA, Tukey, Kruskal-Wallis, Mann-Whitney, and Spearman correlation coefficient were used as appropriated. The statistical significance level of error was considered 0.05.

## Results

From all 72 samples, 50% belonged to men and 50% to women. The mean age of the patients with SCC was 62.4, patients with OLP 41.1, and those with epithelial hyperplasia 30.7. Table 1 shows the patients’ clinical information (age, gender, location). Spearman correlation coefficient revealed a strong relationship between age and staining percentage.

**Table 1:** Summarized information of study groups according to age, gender , and location

**Group**	**Number**	**Mean age**	**Male**	**Female**	**Gingiva**	**Buccal ** **mucosa**	**Tongue**	**Floor of ** **mouth**	**Alveolar mucosa**
Epithelial hyperplasia	24	28.75±11.85	9	15	8	11	2	3	0
Lichen planus	24	34.95±13.13	11	13	9	10	2	3	0
Squamous cell carcinoma	24	63.23±15.37	16	8	1	0	16	3	4

**Figure 1:**
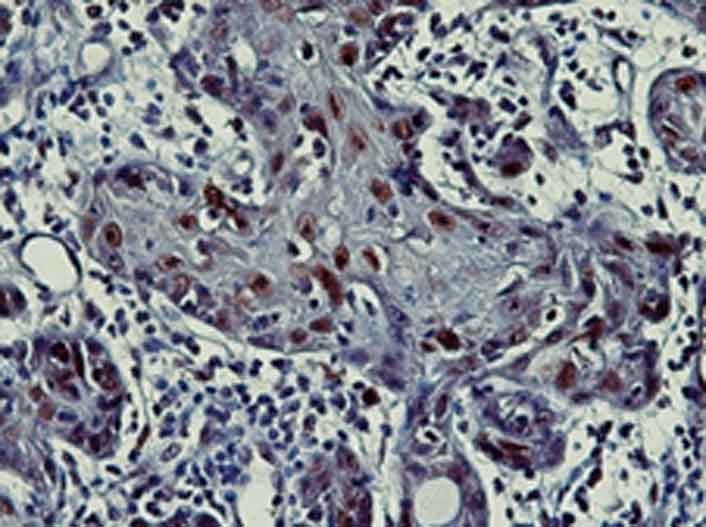
Nuclear p21 immunostaining in oral squamous cell carcinoma

**Figure 2:**
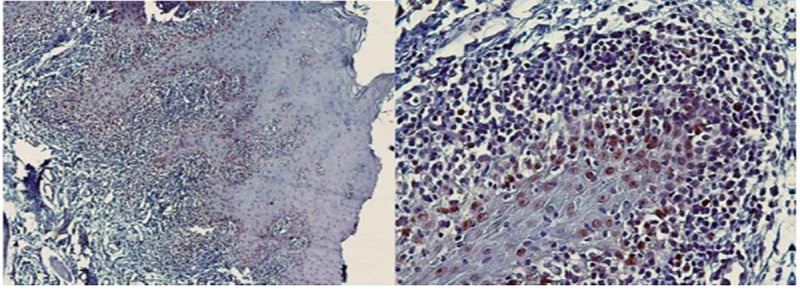
p21 expression in oral lichen planus

**Figure 3:**
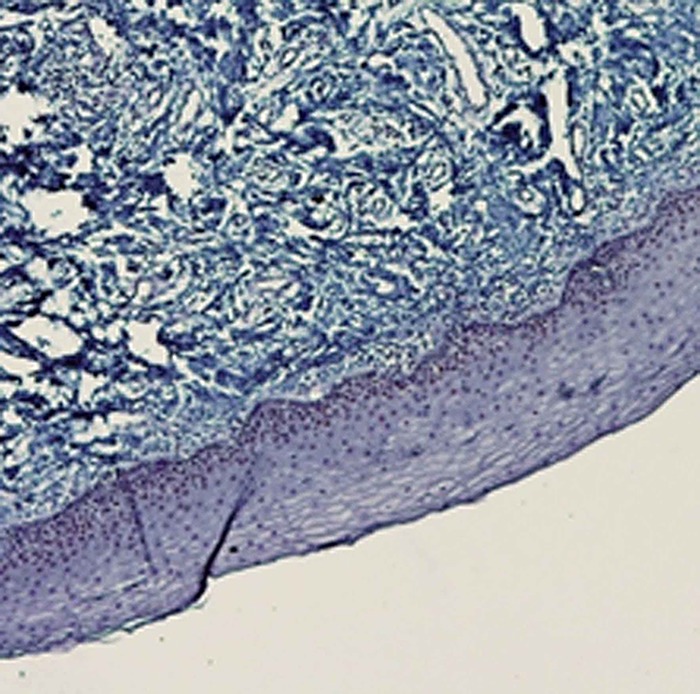
Weak p21 immunostaining in epithelial hyperplasia

The spearman correlation coefficient was calculated to be 0.736 (*p*< 0.001). In each group, the results are summarized in Table 2. The mean percentages of p21-positive cells of SCC (54.5±6.6) (Figure 1) were considerably higher than OLP (32.8±6.08) (Figure 2) and OEH (9.4±3.8) (Figure 3).

**Table 2:** Mean percentages of p21-positive cells in study groups

**Group**	**n**	**Mean** **±** ** SD**	**Minimum**	**Maximum**
Squamous cell carcinoma	24	54.59±6.67	43.80	70.10
Lichen planus	24	32.82±6.08	21.60	41.80
Epithelial hyperplasia	24	9.44±3.81	4	21.2

Comparing the three study groups, Kruskal Wallis test showed that the mean percentages of positive cells and semi-quantitative values have been statistically different (Table 3).

**Table 3:** Semiquantitative results of p21 immunostaining in studied groups

**Group**	**Staining degree**				
	**Negative** **<10%**	**+1** **10-30%**	**+2** **30-50%**	**+3** **>50%**	**Total**
Lichen planus	0	8	16	0	24
Epithelial hyperplasia	13	11	0	0	24
Squamous cell carcinoma	0	0	8	16	24

According to the results of Mann- Whitney test, a statistically significant difference was also observed between OLP and OEH, OLP and OSCC, and OSCC and OEH groups (*p*< 0.001).

The Kruskal Wallis test revealed a significant statistical difference among the groups concerning the intensity of staining, SCC (strong to moderate), OLP (moderate to weak) and Epithelial hyperplasia (weak to negative) (*p*< 0.001). Thus, Mann-Whitney test was performed and the results showed significant statistical correlation between OLP and OSCC and also between OSCC and OEH (*p*< 0.001).

## Discussion

Malignant transformation potential of OLP is rather skeptical.[1, 3, 13] Some researchers believe that atrophic epithelium of lichen planus might be more ready and more sensitive to the carcinogens, resulting in an increased risk of malignant transformation of these lesions.[13-14] Georgakopoulou *et al.* reported that 0-12.5% of oral lichen planus lesions become malignant ,the likely reason that world health organization (WHO) considered it as a potentially malignant disorder.[14] However, the relation between OLP and OSCC is still controversial.[1, 6] In the current study positive p21 staining was observed in all specimens of OSCC and mean percentage of stained cells in OSCC was 54.5%; which was significantly higher than the two other groups (OLP 32.8% and OEH 9.4%). Also, the staining intensity in OSCC was higher than the other groups.

In a study carried out by Pescho *et al.* a reduction in p21 expression from malignant to non-malignant lesions of larynx was reported. P21 expression was also observed in 58.9% of laryngeal SCC samples.[15] In a different study performed on SCC of the esophagus, 73.8% of the samples showed p21 expression. Besides, the level of p21 staining in these tumors was higher than dysplastic lesions and normal mucosa that were studied. Mean percentage of cells stained for p21 in SCC samples was 42.5% and p21 expression was observed in 45% of normal surrounding tissues.[16] Agarwal *et al.* studied p21 in samples of OSCC and observed p21 expression in 68.7% of the samples. Concerning semiquantitative, 20% were grade 1, 22% grade 2 and 28% grade 3.[11] Evaluating p21 expression in OSCC, Ng *et al.* detected p21 in 82.4% of the samples; which was mostly the same as the current study. In their study, 37.5% of the samples showed grade 1 staining (<20%), 38.6% grade 2 (20-50%) and 3.4% grade 3 (>50%).[17] In the study performed by Yanamoto *et al.*, they evaluated p21 expression in OSCC and reported that 53% of the samples showed p21 staining.[18] However, it seems that the p21 staining level is different in human malignancies. For instance, p21 protein expression or p21 mRNA has been detected in SCC of skin, carcinomas of the lungs, cancers of head and neck, and hepatocellular carcinoma while it has been reduced in colorectal and ovarian carcinoma.[18] The differences in the expression of p21, detected in various malignancies, could not be associated with alterations in genes since p21 gene mutation was not observed in many malignancies.[19] p21 binds to a collection of cyclins and CDKs that are effective in cell cycle progression and disables them. The binding affinity of p21 to CDK will increase if it is accompanied by cyclin.[20] Also the inhibitory function of CDKs and p21 are reported to be set stoichiometrically; it causes p21 to activate or deactivate CDKs.[21] According to the previously published studies, p21 is not a permanent CDK inhibitor and it rather affects G1 regulatory cyclins selectively.[20] On the other side, if the proliferating cells are in G1 phase, cell growth would be stopped with low levels of p21; however, if the cells are in synthesis phase (S) they will continue to proliferate until p21 reaches the sufficient level. Thus, in malignancies p21, by itself, is not probably enough to inhibit CDK activity completely and stop the cell growth.[21] Perhaps due to the same reasons, high expression of p21 has been observed in the OSCC samples. The difference in results of the above studies from ours may relate to the difference in type and location of the studied lesions, the number of samples and the method of staining, antibody sensitivity or grading samples. However, further studies on p21 associated with other cell-cycle regulators might be helpful to understand the role of p21 in carcinogenesis.

In this study, the expression of p21 in OLP was significantly higher than that of OEH but it was lower than that of OSCC. Safadi *et al.* studied the p21 expression in OLP and oral mucositis. The mean percentage of stained cells in OLP group (39.9%) was higher than that of oral mucositis (15.06%). Concerning the p21 staining in OLP, the results of their study is similar to that of the current study. Based on their study, it has been suggested that the inflammatory cells and cytokines, found in the surrounding stroma of the lesion, are likely to stabilize p21and p53 proteins and may contribute the developing of the lesion to a tumor.[22] In the study of Hirato *et al.*, p53, p21, TUNEL and all cell proliferation proteins showed increased staining in OLP; which justifies the prolonged maintenance of structural properties of lichen planus and its stable clinical course.[23] In another study, Ilundain *et al.* evaluated the proteins of apoptosis pathway and the cell-cycle regulating proteins in OLP inflammatory cells. They concluded that reduction or loss of apoptosis in inflammatory cells could justify the continued presence of inflammatory infiltration in OLP, molecular abnormalities in epithelial cells, and progress towards developing a cancer.[24] Regarding the fact that the results of various studies show high expression of proliferation markers in OLP,[23, 25-26] increased staining of p21 in OLP is likely to show inactivity of its cell-cycle inhibiting role, which in turn might be the initial genetic event in carcinogenesis.[19] The increased staining of p21 in dysplastic lesions that are transforming to carcinomas has been reported by González-Moles *et al.*,[26] although some reports consider this increase in the p21 expression as a response to hyper proliferative condition.[23, 26]

The results of the current study revealed that among the study groups, the mean age of patients with OSCC was higher than the patients of the other two groups. Also a significant statistical correlation was observed between age and the mean percentages of p21^+^ cells; this result is in line with the results of the study conducted by Ng *et al.*[17] They reported a significant relation between p21 expression in OSCC samples and the patients’ age; the older the patients were the more likely to express p21. In the present study, no statistically significant relation was detected among p21 expression, the location of the lesions, and the patients’ gender; which was consistent with the studies carried out by Ng *et al.* and Yanamoto *et al.*[17-18] Regarding the considerable staining of OLP compared to the OEH, and also based on the results of the present and previous studies, oral lichen planus might be considered among the inflammatory diseases that have the potential to progress to carcinoma.[22, 24] In future studies, evaluating the association of p21 with other cell-cycle regulators may confirm the results of the current study.

## Conclusion

According to the findings of this study, the expression of p21 might be related to the potential carcinogenic transformation of lichen planus to SCC. Follow-up periods are recommended for early diagnosis of the malignant transformations in lichen planus. 
